# Serological and molecular epidemiology of canine adenovirus type 1 in red foxes (*Vulpes vulpes*) in the United Kingdom

**DOI:** 10.1038/srep36051

**Published:** 2016-10-31

**Authors:** David Walker, Seán A. Fee, Gill Hartley, Jane Learmount, Maria J. H. O’Hagan, Anna L. Meredith, Barend M. de C. Bronsvoort, Thibaud Porphyre, Colin P. Sharp, Adrian W. Philbey

**Affiliations:** 1 Royal (Dick) School of Veterinary Studies and The Roslin Institute, University of Edinburgh, Easter Bush, Edinburgh EH25 9RG, UK; 2 Veterinary Sciences Division, Agri-Food and Biosciences Institute, Beltany Road, Coneywarren, Omagh BT78 5NF, UK; 3 Science and Advice for Scottish Agriculture, Roddinglaw Road, Edinburgh EH12 9FJ, UK; 4 National Wildlife Management Centre, Animal and Plant Health Agency, Sand Hutton, York YO41 1LZ, UK; 5 Veterinary Epidemiology Unit, Department of Agriculture, Environment and Rural Affairs, Upper Newtownards Road, Belfast BT4 3SB, UK

## Abstract

Canine adenovirus type 1 (CAV-1) causes infectious canine hepatitis (ICH), a frequently fatal disease which primarily affects canids. In this study, serology (ELISA) and molecular techniques (PCR/qPCR) were utilised to investigate the exposure of free-ranging red foxes (*Vulpes vulpes*) to CAV-1 in the United Kingdom (UK) and to examine their role as a wildlife reservoir of infection for susceptible species. The role of canine adenovirus type 2 (CAV-2), primarily a respiratory pathogen, was also explored. In foxes with no evidence of ICH on post-mortem examination, 29 of 154 (18.8%) red foxes had inapparent infections with CAV-1, as detected by a nested PCR, in a range of samples, including liver, kidney, spleen, brain, and lung. CAV-1 was detected in the urine of three red foxes with inapparent infections. It was estimated that 302 of 469 (64.4%) red foxes were seropositive for canine adenovirus (CAV) by ELISA. CAV-2 was not detected by PCR in any red foxes examined. Additional sequence data were obtained from CAV-1 positive samples, revealing regional variations in CAV-1 sequences. It is concluded that CAV-1 is endemic in free-ranging red foxes in the UK and that many foxes have inapparent infections in a range of tissues.

Infectious canine hepatitis (ICH) is caused by canine adenovirus type 1 (CAV-1) and can cause severe and fatal disease, primarily in domestic dogs and other canids^1^. The disease first came to prominence in the early 20^th^ century, having been described as “epizootic fox encephalitis” in farmed silver foxes, a colour variant of the red fox (*Vulpes vulpes*), in North America^2^. Clinical signs of ICH in dogs and foxes include sudden death, neurological signs and jaundice. Lesions in dogs and foxes include severe necrotising hepatitis, disseminated intravascular coagulation (DIC) and vasculitis, the latter manifesting as encephalitis and glomerulonephritis, caused by viral replication in hepatocytes and vascular endothelial cells^3^,^4^. Mortality from experimental infections is 10–25% in red foxes and 10–30% in dogs^5^.

Most documented information relating to the clinical course of non-experimental ICH is based on reports of natural disease in domestic dogs. However, there have been several documented cases of ICH in free-ranging species, including a grey fox (*Urocyon cinereoargenteus*) in Georgia, USA^6^, and three cases in red foxes in the UK^7^. Furthermore, disease appears not to be restricted to canids; fatal ICH has been suspected in hospitalised black bear cubs (*Ursus americanus*) in the USA^8^ and a captive Eurasian otter (*Lutra lutra*) in Seoul, South Korea^9^.

CAV-1 is widely distributed geographically and evidence of infection has been found amongst canid species worldwide^7^,^10^. The prevalence of antibodies against untyped canine adenovirus (CAV) can be particularly high in free-ranging canid species; for example, 188 of 194 (97%) island foxes (*Urocyon littoralis*) in the Californian Channel Islands^11^ and 54 of 57 (94.7%) of wolves (*Canis lupus*) in Alaska^12^ had antibodies against CAV. In Europe, neutralising antibodies against CAV were detected in sera from 17 of 485 (3.5%) red foxes in Germany^10^ and in tissue fluid extracts from 11 of 58 (19%) red foxes in England and Scotland^7^. However, this seropositivity is not necessarily specific for CAV-1, since there is substantial cross-reactivity between CAV-1 and canine adenovirus type 2 (CAV-2); CAV-2 which is implicated in infectious tracheobronchitis in dogs^1^. Cross-protection between the two viruses is exploited by the routine use of CAV-2-based vaccines in veterinary practice to protect dogs against ICH. There is no current evidence to suggest that CAV-2 is pathogenic in foxes, although Balboni *et al*.^13^ reported detection of CAV-2 by the polymerase chain reaction (PCR) in the faeces of a free-ranging red fox in Italy.

The population of red foxes in the UK is estimated to be approximately 258,000 and this species, the only wild canid in the UK, has adapted well to urban environments^14^. Domestic dogs are therefore likely to come into indirect (or rarely direct) contact with foxes via urine, faeces and infected fomites. In active infections, CAV-1 is shed in urine, faeces and possibly other secretions^1^; therefore, concern has been raised that red foxes may be a wildlife reservoir of CAV-1^7^,^13^, as well as other pathogens, such as *Angiostrongylus vasorum*^15^. In a study of outbreaks of ICH amongst captive juvenile red foxes in wildlife hospitals in the UK, an apparently healthy fox, thought to be shedding infectious CAV-1, was the likely cause of one outbreak^4^. To determine whether foxes are a significant reservoir of CAV-1, and thus a source of infection for dogs and other susceptible species, it is important to determine the prevalence of infection (including inapparent infection) with CAV-1 amongst seemingly healthy foxes in the UK and other countries with high densities of foxes.

To date, the impact and prevalence of CAV-1 in wildlife in the UK has not been fully assessed. This is due, in part, to the practical difficulty in obtaining large sample sizes, an inherent limitation in many wildlife surveys. Furthermore, there is some evidence suggesting that CAV-1 establishes persistent infections in renal tubular epithelial cells in a proportion of domestic dogs and that virus is shed in the urine of these animals for up to 9 months^16^. Therefore, CAV-1 may persist and/or be excreted for varying periods of time at low levels during inapparent infections and it is desirable that more sensitive molecular techniques, such as nested PCR or quantitative real-time PCR (qPCR), are developed for detecting potentially low copy number CAV-1 DNA, so as to minimise false negative results in molecular surveys.

The aim of this study was to examine the role of red foxes in the UK as a wildlife reservoir of CAV-1 and as a potential source of infection for domestic dogs. In this survey, the first in the UK to use molecular methods for the detection and sequencing of CAV-1, we investigated the epidemiology of CAV-1 in red foxes across the UK, comprising Great Britain (GB, which includes England, Scotland and Wales) and Northern Ireland (NI); the occurrence of CAV-1 in red foxes in Wales and NI has not been assessed previously. The survey combined serology, using an indirect enzyme-linked immunosorbent assay (ELISA), and molecular investigations, using a nested PCR. A probe-based qPCR was also developed to estimate viral loads in CAV-1 infected samples, including tissues and urine. Viral loads were quantified for the first time in urine, which is considered to be one of the primary routes of CAV-1 transmission in active infections^1^. We also examined whether red foxes might harbour CAV-2.

## Materials and Methods

### Specimen collection and processing

The project was approved by the Royal (Dick) School of Veterinary Studies (RDSVS), University of Edinburgh, Veterinary Ethical Review Committee (VERC; approval number 103 14). Methods were performed in accordance with relevant guidelines and regulations. All samples in the study were obtained as excess clinical material or as by-products of other activities and events, unrelated to the study. Tissues were obtained from red fox carcasses originating from a variety of sources throughout the UK. A number of animals were collected as a result of land and game management regimes in the Lothians and Borders, Scotland, and in Cumbria, England. Carcasses obtained in this manner were refrigerated at 4 °C or frozen at −20 °C before undergoing post-mortem examination. Foxes were also collected opportunistically from incidents of road traffic accidents (RTAs) or from natural causes of death in Edinburgh and the Lothians and Borders, Scotland. Tissues which had been stored at −80 °C were also utilised from a prior study by Meredith *et al*.^17^.

Other foxes were obtained from wildlife hospitals in Scotland and England, where foxes had died as a result of injury or disease, not including clinical cases of ICH, or had been euthanased by a lethal injection of barbiturate on humane grounds. Carcasses were stored at −20 °C prior to collection for examination. Blood samples included in the study were also obtained from Tiggywinkles Wildlife Hospital, Haddenham, England. Animals which had been euthanased due to disease or injury unrelated to ICH at the RDSVS, were also included in the study. Clinical records were interrogated following each post-mortem examination when possible.

The Animal and Plant Health Agency (APHA), England, Science and Advice for Scottish Agriculture (SASA), Scotland, and the Agri-Food and Biosciences Institute (AFBI), NI, routinely perform post-mortem examinations on fox carcasses for disease surveillance (foxes submitted to AFBI were initially collected by the Department of Agriculture, Environment and Rural Affairs, NI; DAERA)^18^,^19^. Carcasses are provided to these organisations mainly as a result of land and estate management, or sometimes as RTAs or natural causes of death. Blood samples from foxes from these large-scale surveys were subsequently made available for testing, including samples previously utilised by Taylor *et al*.^15^ in an unrelated study. AFBI and SASA provided tissue samples from NI and Scotland, respectively.

When possible, relevant individual data, such as sex, weight and body condition score (BCS), were recorded during post-mortem examination. The age of each fox undergoing post-mortem examination at the RDSVS was estimated on the basis of size, body weight and dentition. To allow data to be merged with that recorded by AFBI and Taylor *et al*.^15^, foxes were assigned to an estimated age class (1 = cub/juvenile, 2 = young adult, 3 = aged adult). Similarly, BCS was scored subjectively (1 = poor, 2 = fair, 3 = good), based on a range of factors, including external and internal fat coverage and muscle mass. Any significant gross lesions were also recorded during the post-mortem examination. The location from where carcasses were collected (grid references, geographical coordinates or place names) were available for most samples. However, the origin of most red foxes admitted to wildlife hospitals could not be traced accurately. Locations, where available, were converted and standardised to longitudes and latitudes to account for the multiple measurement formats. Foxes were also assigned to larger regions, designated NI, Scotland, North England, Midlands and Wales (Central), South East England or South West England.

A range of tissues and samples were collected from foxes in Scotland. The tissues collected, when full sets could be obtained, were blood, liver, kidney, spleen, small intestine, brain, lung, urine and faeces. When the full array of tissues could not be collected, for example due to carcass damage or scavenging, a subset was collected. Only blood, liver and kidney samples were collected from foxes from NI. Samples were collected into polypropylene containers and stored at −20 °C prior to further processing. After collection, blood samples were centrifuged to remove debris and stored at −20 °C. DNA from tissues and urine samples was extracted using the DNA Blood and Tissue Kit (Qiagen, Hilden, Germany), whilst DNA from faecal samples was extracted using the E.Z.N.A Stool DNA Kit (Omega Bio-tek, Norcross, Georgia, USA), then stored at −20 °C prior to further testing.

A total of 154 foxes from across the UK were included in the molecular survey and a total of 469 foxes were included in the serological survey.

### CAV-1/CAV-2 specific PCR protocol

Liver and kidney were classified as the ‘sentinel’ organs for CAV-1 infection in this study; liver is one of the main organs infected during the acute stages of ICH in dogs^1^, and the kidney is considered to be a sentinel organ for ‘chronic’ infection (although it is also infected early in infection) when animals may be shedding virus in their urine^20^. Therefore, only foxes where both liver and kidney were collected were included in the study; DNA extracted from these organs was screened for CAV-1 in all foxes. PCR was performed to screen for both CAV-1 and CAV-2 in the urine and faeces of animals from which these samples were collected, to detect animals which were shedding virus. When a positive result was obtained in DNA extracts from liver and/or kidney, DNA extracts from all other samples collected during the post-mortem examination of the positive animals were tested.

We hypothesised that a low copy number of CAV-1 DNA, particularly in animals without clinical signs or gross lesions typical of ICH, would be present in infected tissues. Therefore, we developed a nested PCR protocol specific for CAV-1 using the CAV-1 reference sequence (EMBL AC_000003.1) (Table 1). An alternative set of nested primers was also developed to detect CAV-2 (EMBL AC_000020.1) (Table 1). The primer sets were designed against a region of the genome to allow discrimination between CAV-1 and CAV-2, which corresponded to putative ORF7 (GenBank Y07760.1).

The PCR first-round reaction mixture for CAV-1 consisted of 35.8 μL H_2_O, 0.2 μL (1 U) GoTaq G2 DNA polymerase (Promega, Madison, Wisconsin, USA), 10 μL 5x Green GoTaq reaction buffer (Promega), 1 μL deoxynucleotide triphosphates (dNTPs; final concentration 200 μM each dNTP), 1 μL CAV_F forward primer (final concentration 200 nM), 1 μL CAV-1_R reverse primer (final concentration 200 nM) and 1 μL DNA. Product (1 μL) from the first round PCR was used as a template for the second round using the second round primer set CAV-1_2F and CAV-1_2R. The CAV-2 reaction was identical except that the first round used the primers CAV_F and CAV-2_R, whilst the second round used CAV-2_2F and CAV-2_2R (Table 1). The reaction conditions were 94 °C for 18 s, 50 °C for 21 s and 72 °C for 1 min, repeated for 40 cycles, followed by one cycle of 72 °C for 5 min.

The specificity of the PCRs using primers designed for each CAV type was verified using clinical case material from fatal ICH cases in foxes^4^ and a commercial vaccine containing CAV-2 (Nobivac DHPPi, MSD Animal Health, Walton, UK). PCR products from these control samples, as well as selected positive study samples, were also sequenced to confirm specificity (Edinburgh Genomics, Edinburgh, UK). The sensitivities of the PCRs for detecting CAV-1 and CAV-2 were assessed using dilutions of plasmid controls containing a CAV-1 or CAV-2 insert (see ‘Quantitative PCR’) and was determined to be less than 10 copies.

Each DNA sample in this study was tested in triplicate, in an attempt to increase rates of detection of very low copy number samples and to negate possible false positive results from contamination. PCR products were loaded on agarose gels stained with SYBR safe DNA stain (Invitrogen, Paisley, UK) and separated by electrophoresis. Amplicons were observed using a G:Box gel viewing system (Syngene, Cambridge, UK). The expected amplicon size of the second round product was 188 base pairs (bp) for both CAV-1 and CAV-2 primer sets. Positive amplicons were confirmed by sequencing (Edinburgh Genomics).

### Additional CAV-1 sequence data

An additional four sets of nested primers, based on genes or transcriptional units (including hexon, fibre, E3 and E4^21^), were designed using the reference CAV-1 genome (EMBL: AC_000003.1) (Table 2). The purpose of these primers was to amplify and analyse sequences in addition to those provided by the CAV-1 detection primer set in positive animals, allowing for comparison of different genomic regions amongst detected sequences from different locations in the UK. The PCR reaction mixture and amplification conditions were the same as those used for screening for CAV-1, except that 2 μL DNA was used (and H_2_O reduced accordingly to 34.8 μL). PCR products were confirmed to be a match for CAV-1 by direct Sanger sequencing (Edinburgh Genomics) using the internal primers. In the case of faint bands on agarose gels, DNA was extracted using the QIAquick Gel Extraction Kit (Qiagen) and cloned using the pGEM-T Easy Vector System (Promega) and DH5α *Escherichia coli* competent cells, before sequencing.

### Quantitative PCR (qPCR)

A CAV-1 specific probe-based qPCR protocol was developed to estimate the viral load of tissues positive for CAV-1 by conventional nested PCR. A dual labelled oligonucleotide probe was used (CAV_probe); this was labelled at the 5′ end with 6-carboxyfluorescein (FAM) and at the 3′ end with 6-carboxytetramethylrhodamine (TAM) (Table 1). The protocol utilised the second round forward and reverse primers designed for the nested PCR protocol (Table 1). DNA (2 μL) was added to a reaction mixture containing 10 μL Brilliant III Ultra Fast qPCR Master Mix (Agilent Technologies, Wokingham, UK), 5 μL H_2_O, 1 μL CAV_probe dual labelled probe (final concentration 500 nM), 1 μL CAV-1_2F forward primer and 1 μL CAV-1_2R reverse primer (each final concentration 500 nM). A CAV-2 specific probe-based qPCR protocol was created using the same conditions and reaction mixture, substituting the CAV-1 second round primers for the CAV-2 primers, CAV-2_2F and CAV-2_2R (Table 1).

DNA samples were repeated within each run in triplicate. Plasmids containing CAV-1 or CAV-2 DNA inserts were used as positive controls for the CAV-1 and CAV-2 qPCR protocols, respectively. Plasmid constructs were created using DH5α *E. coli* competent cells and the pGEM-T Easy Vector System (Promega). The insert DNA was the 674 bp amplicon created with a single round of PCR using the CAV_F and CAV_R primers (Table 1). The DNA templates for both CAV-1 and CAV-2 vectors were from field strains of virus, originating from an outbreak of ICH in foxes in Scotland^4^, and a cultured laboratory stock (University of Glasgow, Scotland, UK), respectively. Plasmid concentrations were estimated using a spectrophotometer (NanoDrop, Thermo Scientific, Wilmington, Delaware, USA). Sonified salmon sperm DNA (50 ng/μL; AppliChem, Darmstadt, Germany) was used to dilute plasmids to their intended calculated dilutions. Control plasmid samples were used within each qPCR run in triplicate and in 10-fold dilutions ranging from 10^6^ to 10^1^ estimated plasmid copy numbers. ‘No template’ control (NTC) samples were also loaded in triplicate.

The qPCR reaction conditions followed a two-step cycle consisting of 95 °C for 10 min, then 45 cycles of 95 °C for 15 s and 60 °C for 60 s. The qPCR cycler (Rotor-Gene, Corbett Life Science, Mortlake, Australia) was set to acquire FAM fluorescence signals during amplification. The resulting data were analysed using Rotor-Gene Q Series software (Qiagen). A threshold value for quantification was determined by automated calculation, requested within the software, which was manually verified and adjusted if appropriate.

### Indirect enzyme-linked immunosorbent assay

An indirect ELISA was developed to assess the anti-CAV antibody status of red foxes in the UK by detection of immunoglobulin G (IgG) in sera or blood from 469 red foxes. The ELISA was optimised on 96-well, flat bottomed, high binding microplates (Greiner Bio-One, Stonehouse, UK) using a chequer board assay following methods adapted from Crowther (2000)^22^. The antigens were prepared as separate supernatants containing whole virus, CAV-1 (ATCC VR293) or CAV-2 (field strain, University of Glasgow); the viruses were propagated in Madin-Darby canine kidney (MDCK) cell cultures. Virus-free supernatant was prepared for use in negative control wells. The preparations were used to coat alternate wells of a microplate at a dilution of 1:80 in carbonate/bicarbonate buffer (Sigma-Aldrich, St Louis, Missouri, USA) in a volume of 100 μL, at 4 °C overnight or at ambient temperature for up to 4 h.

Each well was then washed twice with 250 μL phosphate buffered saline (PBS) containing 0.05% by volume Tween 20 detergent (Sigma-Aldrich) (PBS/0.05% Tween) using an automated microplate washer (Ays Atlantis, Biochrom, Cambridge, UK). The wells were subsequently blocked with 2% bovine serum albumin (BSA; Sigma-Aldrich) diluted in PBS (2% BSA/PBS) at ambient temperature for at least 2 h. Following aspiration of the blocking agent, each serum sample was applied at a dilution of 1:80 in a volume of 100 μL, which was tested in duplicate against the three wells: (1) ‘CAV-1’, (2) ‘CAV-2’ and (3) ‘virus-free’ negative control. Each microplate included CAV-antibody positive and negative control fox sera, which were verified for antibody status with a virus neutralisation test (VNT; see below).

Wells were then aspirated and washed six times over a 1 h period. Horseradish peroxidase (HRP) conjugated goat anti-dog IgG (Abcam, Cambridge, UK) was diluted to 1:1600 in 100 μL 2% BSA/PBS and applied to wells for 30 min. Following four washes over 30 min and aspiration of liquid, secondary antibody was detected using 2,2-azino-bis(3-ethylbenzothiazoline-6-sulphonic acid) substrate (Sigma-Aldrich). The optical density (OD) of each well was measured using a microplate reader (Multiskan Ascent, Thermo Scientific, Waltham, Massachusetts, USA) at a wavelength of 405 nm (OD_405_).

The mean OD_405_ reading for all samples was corrected for background reactivity by subtraction of the mean OD_405_ recorded from the virus-free control wells for each sample, and also corrected by a calculated ‘inter-plate variability factor’. This was calculated as the percentage difference between the OD_405_ of the positive control serum against CAV-1 or CAV-2 on the designated reference plate (arbitrarily assigned as the first test plate) compared to the positive control sample on the current test plate.

An animal was declared as positive for antibodies reactive against CAV-1 or CAV-2, based on OD_405_ cut-off values calculated separately for CAV-1 and CAV-2. The cut-off values for IgG positivity were estimated using receiver operating characteristic (ROC) curves to provide an objective method of ELISA cut-off estimation. Data used for the ROC curves were OD_405_ values recorded from a sub-population of CAV-1 and CAV-2 antibody positive and negative control sera determined by a CAV VNT (see below). The ROC curves were computed using the *pROC* package^23^ within R Studio version 0.99, running R version 3.2.4 (R Studio, Boston, Massachusetts, USA)^24^. The OD_405_ cut-off values for CAV-1 (0.2475) and CAV-2 (0.2500) were estimated from the ROC curves using the ‘ROC01’ method implemented by the *OptimalCutpoints* package^25^,^26^. A binary value was assigned to the final OD_405_ of each animal to indicate that they were positive or negative for CAV IgG (Supplementary Dataset S1).

### Virus neutralisation test

A CAV VNT was developed using non-haemolysed control sera, which consisted of canine sera obtained from the RDSVS and fox sera obtained from Tiggywinkles Wildlife Hospital. These sera were suggestive of being negative or positive for CAV IgG in the CAV ELISA (i.e. subjectively very low or moderate-to-high OD_405_) prior to the determination of the estimated absolute cut-off OD_405_ for the CAV ELISA (Supplementary Dataset S2).

The VNT was optimised following methods adapted from those described by Loeffen *et al*.^27^. Serum was diluted two-fold on 96-well tissue culture plates, from a dilution of 1:5 to a final dilution of 1:5120. Sera were tested in duplicate against both CAV-1 and CAV-2 at 100 50% tissue culture infectious doses (TCID_50_)/50 μL. Sera were incubated with virus for 1 h at 37 °C before the addition of ~8 × 10^4^ MDCK cells in 100 μL cell culture medium. The virus load was verified by back titration of 100 TCID_50_ CAV-1 or CAV-2 in 10-fold dilutions ranging from 10^−1^ to 10^−5^. Plates were read on day 5 post-infection; wells in the plates were assigned as positive or negative for cytopathic effect (CPE). The antibody titre against CAV-1 or CAV-2 for each serum sample was calculated as the average titre within each duplicate. Absolute negative sera (titre = 0) were distinguished from CAV-1/CAV-2 positive sera to identify control sera for calculation of the cut-off OD_405_. The cut-off was then verified by ‘back-analysis’ of the ELISA binary result for the VNT controls.

### Statistical analyses

A generalised linear model (GLM) was used to evaluate the association of the CAV IgG status (positive or negative) of the sampled red foxes with both demographic and environmental variables. The CAV IgG status used in the model was based on the estimated cut-off value applied to the mean OD_405_ against CAV-1 of sera in the ELISA. Individual descriptors included sex, age class, BCS and region of capture/collection. On the basis of the spatial location of the foxes, we extracted information for several habitat and environmental variables, considered to be potentially influential to the likelihood of CAV-1 infection, from freely available digital environmental data (Table 3). We considered that human population density and land cover may be associated with the population density of red foxes and the likelihood of contact between individuals, whereas maximum and minimum temperature, relative humidity and precipitation may also be associated with individual immune status. Both human density and variables were log_10_(x + 1) transformed to normalise their distributions.

All explanatory variables were screened for missing values, so that foxes were only included in the model if all variables were known, and then evaluated for strong collinearity using bivariable plots for all continuous variables. Several of the environmental variables were strongly correlated with each other and with ‘region’. A backward stepwise elimination process was used to retain covariates (along with biologically plausible two-way interactions) in the multivariable logistic regression model. Variables were retained in the multivariable model if they confounded other variables or if they significantly improved model fit at an α-level < 0.05 using the likelihood ratio test (LRT). Akaike’s information criterion (AIC) was used to determine which combination of variables best explained the data with the minimal number of covariates (i.e. the most parsimonious model). The coefficient estimates and standard errors were monitored during model selection for evidence of instability. Evidence of overfitting of the model was evaluated using a bootstrapping approach, as suggested by Harrell *et al*.^28^, and examination of shrinkage of the slope and intercept parameters. An automated step-wise selection based on minimising the AIC was also run for comparison.

Goodness-of-fit was ensured by the calculation and plotting of several diagnostic measures (including deviance Δ*D*, Pearson’s chi-square Δχ^2^ and influence Δβ) against the predicted probabilities, as suggested by Hosmer and Lemeshow (2000)^29^. The goodness-of-fit of the overall model was further assessed using the area under the curve (AUC) of the ROC curve created from the model. Model sensitivity and specificity were estimated at the proposed thresholds and their 95% confidence intervals were computed using 2000 bootstrap iterations.

To identify the presence of residual spatial autocorrelation in the data, a binned omni-directional semi-variogram^30^ was constructed over the model’s residuals. Semi-variance was computed over distances of up to 10 km and compared to those obtained from a series of 999 Monte Carlo simulations.

**Figure 1:**
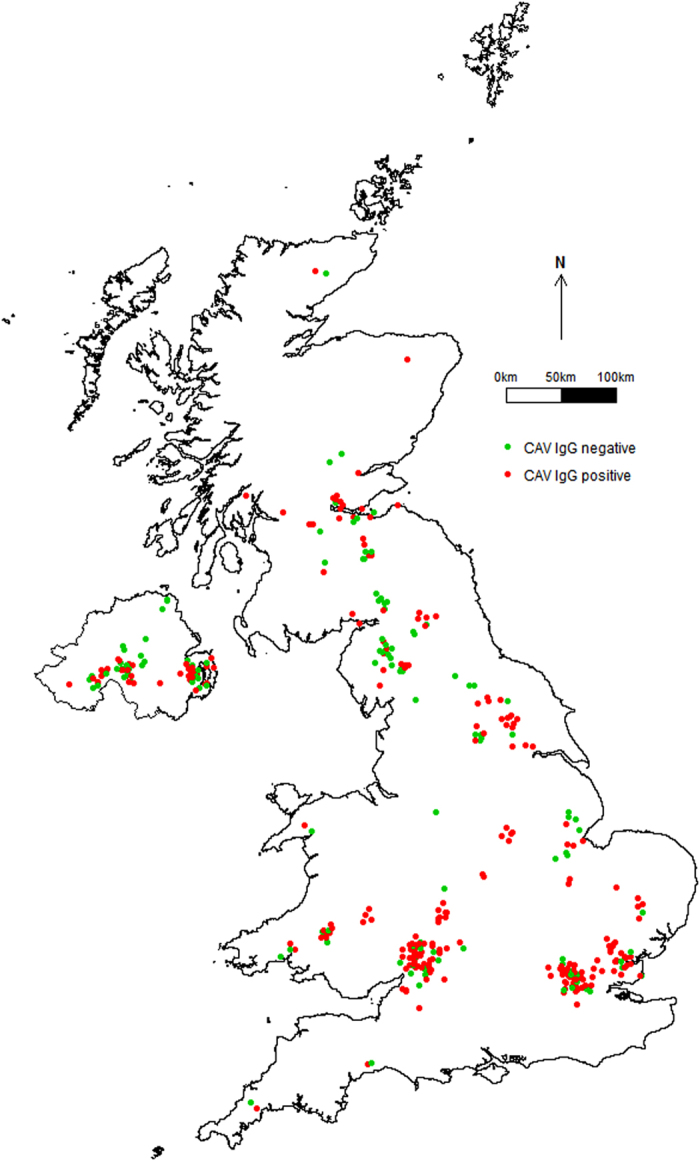
Spatial distribution of red foxes sampled in the United Kingdom (*n* = 387), according to canine adenovirus (CAV) IgG status. Jittering of data points was applied to improve the differentiation of overlapping data points. The map was created in R^24^,^33^.

Analyses were carried out in R^24^. The R packages *ggplot2*^31^ and *rgdal*^32^ were used to visualise the distribution and IgG status of foxes tested by the CAV ELISA (Fig. 1), using a map of the UK obtained from the Database of Global Administrative Areas version 2.8^33^. Other mapping and environmental data extraction procedures were carried out using the *maptools*^34^ and *raster*^35^ packages, respectively. Final model selection was verified by computing AICc and ΔAIC using the R package *AICcmodavg*^36^. Goodness-of-fit procedures were carried out with the *pROC*^23^ and *LogisticDx*^37^ packages, whereas the binned omni-directional semi-variogram was computed using the *geoR* package^38^.

## Results

### Prevalence of CAV-1 and CAV-2 by PCR

CAV-1 DNA was detected by nested PCR in tissue DNA extracts from 29 of 154 (18.8%, 95% confidence interval, CI 13.2–26.1%) foxes across the UK. One additional fox was negative for CAV-1 by PCR in DNA extracts from liver and kidney, but was positive in the spleen, which was tested non-routinely prior to confirmation of liver and kidney negativity. Urine and faeces from all foxes, when available, were tested for both CAV-1 and CAV-2. Three of 17 (17.6%, 95% CI 4.7–44.2%) urine samples available for testing were positive for CAV-1 by PCR. All foxes with positive results in urine were also positive for CAV-1 by PCR in liver and kidney. None of 19 foxes were positive for CAV-1 by PCR in faeces and no animals were positive for CAV-2 in either faeces or urine.

### Distribution and viral load of CAV-1 in fox samples

No animals had gross lesions suggestive of ICH at post-mortem examination. However, because of the nature of the samples, tissues from most foxes were not suitable for histological processing and examination. Two foxes exhibited mild jaundice at post-mortem examination, but both were negative by PCR for CAV-1.

For positive animals, all the available collected samples were screened for the presence of detectable CAV-1 (PCR results for individual positive animals are summarised in Supplementary Table 1). The tissue distribution of CAV-1 and quantification by qPCR, among these positive cases, are summarised in Table 4.

### Prevalence of IgG antibodies against CAV-1

Across the UK, the sera of 302 of 469 (64.4%, 95% CI 59.8–68.7%) red foxes were estimated to be positive for IgG antibodies reactive against CAV-1 by ELISA. Amongst CAV-1 PCR positive foxes subjected to serological testing, 20 of 25 (80%, 95% CI 58.7–92.4%) foxes with blood available for serology had IgG reactive against CAV-1 by ELISA. Amongst CAV-1 PCR negative foxes, 48 of 103 (46.6%, 95% CI 36.8–56.7%) were positive for IgG reactive against CAV-1. IgG antibodies reactive against CAV-2 by ELISA were detected in 264 of 469 (56.3%, 95% CI 51.7–60.8%) foxes. The cross-reactivity between CAV-1 and CAV-2 was high (84.1%, 95% CI 79.4–87.8%). Of foxes positive for CAV IgG, 254 of 312 (81.4%) were estimated to have IgG reactive to both CAV-1 and CAV-2, whereas 48 of 312 (15.4%) animals had IgG reactive to only to CAV-1. Only 10 of 312 (3.2%) animals had antibodies which were reactive only to CAV-2.

Amongst the subset of red foxes with associated spatial data, included in the multivariate model, 257 of 387 (66.4%, 95% CI 61.4–71.1%) foxes were seropositive for CAV. Initial plotting of the spatial distribution suggested a possible north/south trend in prevalence, with a higher prevalence in the south of the UK (Fig. 1). Looking at the regional differences, 37 of 72 (51.4%, 95% CI 39.4–63.2%) in NI were CAV seropositive, compared to 220 of 315 (69.8%, 95% CI 64.4–74.8%) in GB; this difference was statistically significant (χ^2^ = 8.14, df = 1, *p* = 0.004). This initial difference was largely explained by a significant difference in the age structure of the foxes in the samples from NI and GB (χ^2^ = 19.89, df = 2, *p* < 0.001).

In total, 11 variables were tested for association with the odds of foxes being CAV seropositive, including region, age, sex, BCS and the seven environment and habitat variables (Table 3). The final model included the age and sex of the sampled foxes, as well as the mean monthly maximum temperature recorded at the capture/collection site. Compared to being a juvenile fox, being an aged adult fox significantly increased the odds of being CAV seropositive by a factor of 2.75 (95% CI 1.11–6.93), whereas young adult foxes were not significantly more likely to be CAV seropositive (odds ratio, OR, 1.51, 95% CI 0.67–3.46).

There was no statistically significant difference between male and female foxes in the likelihood of being CAV seropositive (OR for male compared to female foxes 0.70, 95% CI 0.45–1.10), but this variable was retained in the model to adjust for potential confounding. The mean monthly maximum temperature was the most influential variable in the model, accounting for 60% of the deviance explained. For each degree increase in the mean monthly maximum temperature, the odds of being CAV seropositive increased by a factor of 1.45 (CI 1.23–1.71). Adding the variable ‘region’ with or without the temperature variable did not improve the fit of the model, confirming that the effect of temperature was not related to the spatial location of the foxes.

The AUC of the final model was 0.68 (95% CI 0.62–0.73) and the index corrected shrinkages for the intercept and slope were 0.047 and 0.922, respectively. There were no overly influential covariate patterns observed in subsequent model diagnostic plots and there was no residual spatial clustering detected when semi-variance was evaluated. This suggests that the model has reasonable explanatory power and little of evidence of overfitting.

### CAV-1 sequence analysis

Sequences obtained using the additional sequence primers (Table 2) were submitted to GenBank under accession numbers KU755693 to KU755761. Sequences obtained using the detection primer set were not submitted due to their relatively short length (<200 bp). The sequences obtained using all primer sets shared 99–100% identity with the reference genome (EMBL AC_000003.1). However, all sequences obtained from the hexon region, using the CAV-1_hex primer set (*n* = 17), were identical to the reference genome in all foxes (GenBank accession numbers KU755693 to KU755701).

**Figure 2:**
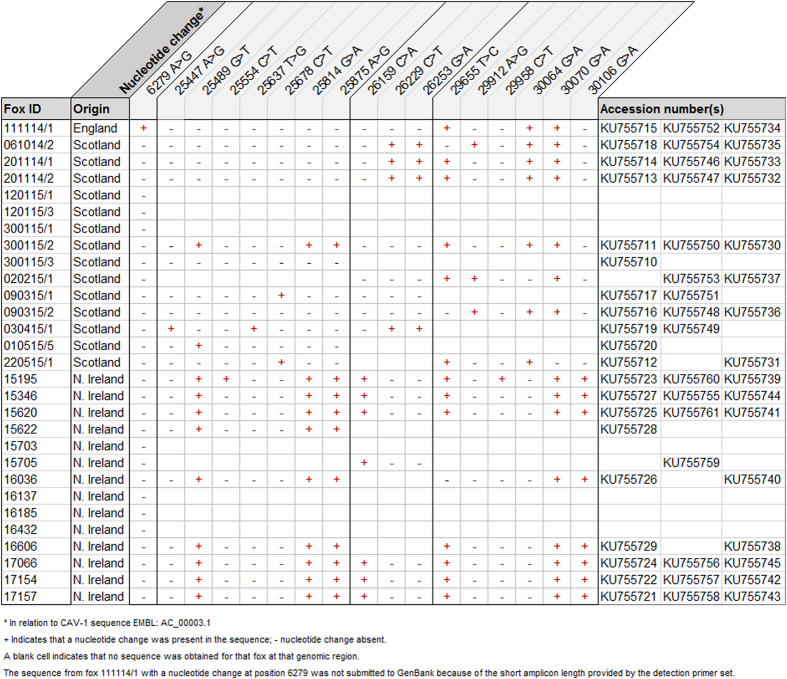
Summary of the common, single nucleotide changes among sequences obtained from foxes in Great Britain (England and Scotland) and Northern Ireland, and the associated GenBank accession numbers.

Single nucleotide changes which were unique to foxes from GB or NI were identified in other amplified genomic regions, in addition to several single nucleotide changes, which were present mostly, but not solely, in one of the populations; these are summarised in Fig. 2. These changes may represent single nucleotide polymorphisms (SNPs). Single nucleotide changes in relation to the CAV-1 reference genome, which were present in two or less foxes from which sequences were obtained, were also identified (Fig. 2); these were verified by repeat sequencing in both forward and reverse directions to negate sequencing errors.

## Discussion

This study has shown that 18.8% of red foxes from across the UK, all of which were free from gross evidence of ICH on post-mortem examination, were positive for CAV-1 sequences by PCR, consistent with inapparent infection. A range of tissues were infected and 50% of urine samples from CAV-1 infected foxes were also positive for CAV-1 by PCR. The majority of foxes with detectable CAV-1 DNA also had antibodies against CAV, which strongly suggests that CAV-1 may establish persistent infections in foxes. The majority (64.4%) of red foxes from across the UK had antibodies against CAV, indicating that the red fox is likely to be a significant wildlife reservoir for CAV-1 in the UK.

It is not possible to estimate how many CAV-1 infections in free-ranging foxes actually result in clinical ICH and how many foxes will die from the disease, despite mortality being reported to be up to 25% in experimental infections in captive foxes^5^. Most cases of ICH are likely to go unnoticed (or undiagnosed) in free-ranging animals and many affected foxes are likely to die underground in their dens, so that the carcasses are never found and examined. In the present study, all of the CAV-1 infections were inapparent, suggesting that a proportion of red foxes do not develop fatal ICH, but instead become persistently infected. Given the high number of red foxes which were estimated by ELISA and PCR to have been exposed to CAV-1, it appears that many animals are subclinically infected or develop only mild ICH, from which they recover. It is possible that some foxes are protected from CAV-1 by maternal antibodies when young and subsequently develop acquired immunity through intermittent exposure to CAV-1 from infected conspecifics.

Aged adult foxes were significantly more likely to have antibodies against CAV than juvenile foxes, consistent with a longer period of potential exposure to CAV-1. Given that protective antibody titres towards CAV appear to be maintained in most dogs which have not been vaccinated for more than 3 years^39^, and that the mean lifespan of free-ranging, urban red foxes in the UK was as low as 2.1 years for subordinate individuals in one study^40^, it is likely that infection and exposure to CAV-1 promotes lifelong persistence of antibodies against CAV in most foxes. CAV-1 appears to be highly prevalent in the UK. Therefore, initial infection may occur early in life following the waning of any maternal immunity and these surviving juvenile animals then progress to adulthood. In the present study, 20% of foxes were positive for CAV-1 by PCR, but did not possess detectable antibodies against CAV. These may have been only recently exposed to CAV-1, and thus may not yet have developed IgG, or this could be a consequence of the high ELISA cut-off value in this study. It is notable that 80% of foxes which were PCR positive for CAV-1 possessed detectable antibodies against CAV; this suggests that CAV-1 possesses effective mechanisms to evade the immune system and persist in the foxes. However, it should be noted that cell-mediated immunity was not measured in this study.

The seropositivity in this study (64.4%) is higher than has been previously estimated in the UK^7^. This finding is likely to be a result of the large sample size, a more sensitive serological diagnostic test and the use of blood instead of tissue fluids, rather than a temporal increase in seroprevalence since the time of the survey by Thompson *et al*.^7^. The relatively high CAV seroprevalence in the present study should not be viewed as an unusual finding, since it is consistent with a seroprevalence of 59.7% reported in red foxes in Norway^41^; furthermore, considerably higher estimates of seroprevalence of CAV have been estimated in other canid species^11^,^42^.

Geographical differences in CAV seroprevalence were observed, with red foxes from southern regions of GB being more likely to be seropositive than those from the north; this association was related to mean monthly maximum temperature. Such an association is most likely to be related to an unobserved variable, such as fox density; fox populations and thus densities are estimated to be higher in the south of GB^14^. Together with the effect of temperature and longitude (possibly related to an unobserved variable, such as population density), the difference in the age structure of the sampled fox populations between NI and GB may account for the observed lower seroprevalence of CAV in red foxes in NI. The study population was largely obtained through convenience sampling and, although it represents a relatively large sample of foxes from across the UK, inferences from the analyses may be biased. However, it is unlikely that biases through convenience sampling are associated with CAV infection, so can be controlled for in the modelling process. Alternatively, it is possible that the population structure and dynamics of red foxes in Ireland (NI and the Republic of Ireland) are different to those in GB. However, detailed studies on the structure of the Irish red fox population have not been published to date.

The high serological cross-reactivity between CAV-1 and CAV-2 is somewhat, but not wholly, addressed using inferences from the associated molecular survey and from clinical evidence. We found that numerous foxes in the UK had evidence of infection with CAV-1 by PCR. However, CAV-2 was not detected in the urine or faeces of any red foxes tested. In addition, although there is a single report of a CAV-2 sequence being detected by PCR in the faeces of a red fox^13^, there is no definitive evidence to suggest that CAV-2 is a frequent infection or causes disease in this species. Therefore, red foxes are unlikely to be a major transmitter of CAV-2 in the UK.

Although persistent excretion of virus in urine has been observed in dogs which have recovered from clinical disease after experimental infection with CAV-1^16^, it has not been demonstrated previously that free-ranging foxes with molecular evidence of inapparent infection with CAV-1 in tissues also shed CAV-1 in urine. Despite the low sample size, it should be noted that the viral load of CAV-1 was relatively high in urine (Table 4), suggesting that this may be an important sample to screen in epidemiological studies of CAV-1 and possibly as a monitoring tool in wildlife hospitals which regularly admit red foxes. Adenoviruses are considered to be moderately resistant in the environment^1^. Therefore, a susceptible red fox, dog or other susceptible species which comes into contact with the recently voided urine of a CAV-1 shedding fox is likely to be at risk of infection.

Although Balboni *et al*.^13^ found that 2 of 32 (6.3%) red foxes in Italy excreted CAV-1 in faeces, there was no evidence of excretion of CAV-1 in faeces from red foxes in our study. Faeces generally are not considered to be a primary route of excretion of CAV-1 in animals which have recovered from ICH^1^. However, it is possible that the detection of CAV-1 may have been limited as a consequence of the small number of samples screened. Although faeces can contain PCR inhibitors, adenoviruses have been successfully detected in faecal samples in other studies^13^,^43^,^44^ and the faecal DNA extraction kit selected was chosen to improve the removal of PCR inhibitors.

In addition to liver and kidney, the spleen, lung and brain were shown to be infected with CAV-1 in some red foxes in the present study. Therefore, CAV-1 appears to establish possibly persistent infections in a range of tissues corresponding to those most often affected during the clinical course of severe ICH^4^. There would be value in testing gastrointestinal and oral lymphoid tissues for evidence of CAV-1, particularly since oral lymphoid tissues, have been shown to be sites of adenovirus persistence in humans, associated with intermittent excretion in faeces in humans and non-human primates^43^,^44^.

Some differences in viral load were found between sample types by qPCR; for example, relatively high loads were observed in urine, kidney and liver compared to the brain, in which viral loads were too low to be quantified. However, due to the small number of samples tested from these sites, it was not possible to apply statistical analysis to the data. In view of the low viral loads in some tissues, a highly sensitive molecular assay, such as nested PCR, is necessary to estimate the true prevalence of inapparent infections with CAV-1.

Since the present study has provided evidence for inapparent infections in red foxes, further molecular experimental studies are required to investigate the pathogenesis of persistent CAV-1 infections and the viral or host mechanisms which underlie this. Some similarities are evident between inapparent CAV-1 infections in red foxes and human adenoviruses^43^,^45^,^46^. However, it is notable that, in this study, CAV-1 DNA was detected in a range of functionally distinct lymphoid and non-lymphoid tissues, in otherwise healthy animals. Moreover, in humans, it is unclear whether, in some cases, systemic disease is a result of recrudescence of a persistent infection or as a result of infection in transplanted tissues (e.g. in transplant recipients)^47^,^48^. Whether similar recrudescence events can occur in persistently infected non-human species is not known, but would be of particular importance when, for example, a free-ranging animal is brought into captivity (i.e. hospitalised), which may result in considerable individual stress. Some CAV-1 proteins in the early expressed (E) regions have low similarity to E gene products of human adenoviruses^21^, so CAV-1 may possess unique evasion mechanisms.

It was not possible to determine the duration of CAV-1 persistence in red foxes, or the cell type(s) that support persistence. Suitable *in vitro* models using canine or vulpine cell lines could be used to investigate the pathogenesis of CAV-1 persistence. It is unclear whether CAV-1 detected by PCR in the organs of carrier foxes is viable and capable of lytic infections in permissible cell lines; this could be investigated using freshly harvested tissues.

This study also presents evidence of genetic variation in the sequences of CAV-1 in foxes from different regions in the UK. This manifests as single nucleotides changes (or possible SNPs) in different regions of the CAV-1 genome, with some nucleotide variants appearing to be unique to foxes from GB or NI. Genetic variation may be a result of genetic drift caused by the disruption of gene flow between GB and Ireland, which are geographically separated by the Irish Sea. Such spatial variation in the CAV-1 genome has not been reported previously. There may be a variable rate of divergence in different regions of the CAV-1 genome, since sequences obtained from the hexon region were invariant, whereas others were more variable. The sequences from the possible multiple CAV-1 field strains circulating in red foxes in the UK are different to those which historically have been detected in dogs in the UK and Europe. The reference genome for CAV-1 was sequenced from a field strain isolated from a dog in the UK in 1996^21^. Two CAV-1 sequences reported in dogs from Italy from 2012 were identical to this reference sequence, although only the CAV-1 E3 region was sequenced in these cases^49^. CAV-1 molecular epidemiological studies should be extended to dogs and red foxes in other countries, and other genomic regions should be sequenced to allow direct comparisons with the CAV-1 sequences obtained in the present study.

## Conclusion

ICH is now generally considered to be a relatively uncommon disease in domestic dogs in areas where vaccination is performed routinely. However, because a high proportion of red foxes in the UK are inapparently infected with CAV-1 and, in some cases, CAV-1 is shed in the urine, it is recommended that all dogs continue to be routinely vaccinated against ICH (using CAV-2-based vaccines). Consideration of current wildlife reservoirs of disease should also be strongly considered in the planned or potential reintroductions of susceptible free-ranging carnivores to historical habitats, for example the debated reintroduction of wolves into Scotland^50^,^51^, which may become an additional reservoir of CAV-1 in the UK. Consideration should also be given to the management and vaccination of red foxes in wildlife rescue centres to prevent outbreaks of ICH^4^. Small animal veterinarians should be aware that there is a risk of infection in unvaccinated animals, and ICH should be suitably considered in the differential diagnoses of critically unwell, unvaccinated dogs and other susceptible species with supporting clinical history. It is likely to be both practically and financially unfeasible to attempt a disease eradication programme for CAV-1 in red foxes in the UK.

As the only free-ranging canid species in the UK, the red fox is deemed to be the primary wildlife reservoir of CAV-1. Other canid species may be the primary, or an additional reservoir, of the virus in other countries, and sensitive molecular methods should be employed to investigate this. Further molecular studies investigating the pathogenesis of CAV-1 should specifically aim to identify the viral mechanism(s) which may permit CAV-1 to persist in the tissues of infected hosts.

## Additional Information

**How to cite this article**: Walker, D. *et al*. Serological and molecular epidemiology of canine adenovirus type 1 in red foxes (*Vulpes vulpes*) in the United Kingdom. *Sci. Rep*. **6**, 36051; doi: 10.1038/srep36051 (2016).

**Publisher’s note:** Springer Nature remains neutral with regard to jurisdictional claims in published maps and institutional affiliations.

## Supplementary Material

Supplementary Information

Supplementary Dataset S1

Supplementary Dataset S2

## Figures and Tables

**Table 1:** Summary of the detection primers specific for canine adenovirus type 1 (CAV-1) and type 2 (CAV-2) and the labelled oligonucleotide probe.

Oligonucleotide name	Description	Nucleotide sequence (5′–3′)a,^b^	Nucleotide position on CAV-1/CAV-2 genome^a^,^b^
CAV_F	CAV-1 and CAV-2, forward, 1^st^ round	TAYTCATACATTTCATTGGAG	6184–6204 (CAV-1) 6272–6292 (CAV-2)
CAV_R	CAV-1 and CAV-2, reverse 1^st^ round	GCAGAAATMCCACCCGTG	6840–6857 (CAV-1)^1^ 6928–6945 (CAV-2)^2^
CAV-1_2F	CAV-1, forward, 2^nd^ round	ATTATCGTGTTTAGATGGGGGGC	6214–6236
CAV-2_2F	CAV-2, forward, 2^nd^ round	AGGATGGTACTTAGGTGGTGTGT	6302–6324
CAV-1_R	CAV-1, reverse, 1^st^ round	TACGTGCCTAGCAAAGATTACAGA	6735–6758
CAV-2_R	CAV-2, reverse, 1^st^ round	TACGTGCCTGCCAAGAGTTACGAG	6823–6846
**CAV-1_2R**	CAV-1, reverse, 2^nd^ round	GTAACAGCCCAGCTAGTTAACAAG	6378–6401
CAV-2_2R	CAV-2, reverse, 2^nd^ round	GTAACAGCCCAGTTGGTAAACAAA	6466–6489
CAV_probe	Probe, CAV-1 and CAV-2	FAM-GAGCTGATGGTTGGACGCTGGAAGAC-TAM	6289–6314 (CAV-1) 6377–6402 (CAV-2)

^a^EMBL AC_000003.1.

^b^EMBL AC_000020.1.

**Table 2:** Summary of additional sequencing primers for CAV-1.

Oligonucleotide name	Description	Nucleotide sequence (5′–3′)^b^	Target(s)^a^,^b^	Nucleotide position on CAV-1 genome^b^
**CAV-1_hex_F**	Forward, 1^st^ round	CATGGCACACAACACAGC	Hexon [partial]	18475–18492
**CAV-1_hex_2F**	Forward, 2^nd^ round	GTAAATGACCAGTCCTTTGC	Hexon [partial]	18524–18543
**CAV-1_hex_R**	Reverse, 1^st^ round	GAATTGTTATGCTGGTGACC	Hexon [partial]	19070–19089
**CAV-1_hex_2R**	Reverse 2^nd^ round	AAGTGGTAGCCTTGATAGC	Hexon [partial]	18942–18960
**CAV-1_E3_F**	Forward, 1^st^ round	CTCTGTCTCTCCAATGGC	Putative orf 23/Early E3 22.1 kDA glycoprotein Q96688 [partial], orf 24, orf 25 [partial]	25293–25310
**CAV-1_E3_2F**	Forward, 2^nd^ round	ACTATCATGCCGCTGAAC	Putative orf 23,/Early E3 22.1 kDA glycoprotein Q96688 [partial], orf 24, orf 25 [partial]	25396–25413
**CAV-1_E3_R**	Reverse, 1^st^ round	GAGGCGAGATATTCACAGC	Putative orf 23/Early E3 22.1 kDA glycoprotein Q96688 [partial], orf 24, orf 25 [partial]	26019–26037
**CAV-1_E3_2R**	Reverse, 2^nd^ round	GGGGCGTCATATGGATACAC	Putative orf 23/Early E3 22.1 kDA glycoprotein Q96688 [partial], orf 24, orf 25 [partial]	25929–25948
**CAV-1_fib_F**	Forward, 1^st^ round	CCGTGTATCCATATGACGC	Fibre [partial]	25927–25945
**CAV-1_fib_2F**	Forward, 2^nd^ round	CTCTGGCTGTGAATATCTCG	Fibre [partial]	26014–26033
**CAV-1_fib_R**	Reverse, 1^st^ round	TTGCTGGAGGTTGAACTGC	Fibre [partial]	26490–26508
**CAV-1_fib_2R**	Reverse, 2^nd^ round	TAGTACGGTGAGACCCGGAC	Fibre [partial]	26455–26474
**CAV-1_E4_F**	Forward, 1^st^ round	GCCCACTGTGACTAGAAAGC	Putative orf29 Q96692 [partial] and orf30 Q96693	29544–29563
**CAV-1_E4_2F**	Forward, 2^nd^ round	CGACACAAATCTGTCTCGC	Putative orf29 Q96692 [partial] and orf30 Q96693	29615–29633
**CAV-1_E4_R**	Reverse, 1^st^ round	GCTGATTTCCTGAGACGC	Putative orf29 Q96692 [partial] and orf30 Q96693	30283–30300
**CAV-1_E4_2R**	Reverse, 2^nd^ round	GAGACTTCATTCTCGACAGC	Putative orf29 Q96692 [partial] and orf30 Q96693	30207–30226

^a^Morrison *et al*.^21^.

^b^EMBL: AC_000003.1, GenBank Y07760.1.

**Table 3:** Additional explanatory variables used in the regression model development with data source.

Variable	Unit	Year	Spatial resolution	Name and source of data	Reference
Human population density	km^−1^	2011	1 km	UK gridded population, CEAH	Reis *et al*.^52^
Relative humidity	%	2011	5 km	Gridded observation data (UKCP09), Met Office	Perry and Hollis^53^
Total annual rainfall	mm	2011	5 km	Gridded observation data (UKCP09), Met Office	Perry and Hollis^53^
Mean monthly maximum temperature	°C	2011	5 km	Gridded observation data (UKCP09), Met Office	Perry and Hollis^53^
Mean monthly minimum temperature	°C	2011	5 km	Gridded observation data (UKCP09), Met Office	Perry and Hollis^53^
Altitude	m	—	1 km	The Shuttle Radar Topographic Mission (SRTM) digital elevation data, CGIAR-CSI	Jarvis *et al*.^54^
Land cover class	—	2012	250 m	Corine Land Cover European database, Copernicus Land Monitoring Services	European Environment Agency^55^

**Table 4:** Distribution of CAV-1 infection among tissues/samples in foxes positive for CAV-1 by PCR, and estimation of viral load (genome copies per μL) by qPCR.

	Liver	Kidney	Blood	Spleen	Brain	Lung	GIT	Urine	Faeces
Percentage (%) of CAV-1 PCR positive foxes	85 (*n* = 29)	61.9 (*n* = 29)	27.3 (*n* = 22)	83.3 (*n* = 6)	62.5 (*n* = 8)	33.3 (*n* = 9)	0 (*n* = 4)	50 (*n* = 6)	0 (*n* = 6)
Mean CAV-1 genome copies/μL	1.15 × 10^3^	6.50 × 10^3^	1.14 × 10^1^	5.45 × 10^2^	BLD	1.43 × 10^1^	—	1.42 × 10^4^	—
Standard deviation	2.66 × 10^3^	2.30 × 10^4^	2.27 × 10^1^	1.06 × 10^3^	—	1.69 × 10^1^	—	2.41 × 10^4^	—

GIT, gastrointestinal tract; BLD, below the limits of detection.

## References

[b1] Decaro N., Buonavoglia C., Eatwell K., Erdélyi K. & Duff J. P. Adenovirus infections. In Infectious Diseases of Wild Mammals and Birds in Europe 1st edn (eds Gavier-Widén D., Duff J. P. & Meredith A.) Ch. 14, 210–218 (Wiley-Blackwell, 2012).

[b2] Green R. G., Zeigler N. R., Green B. B. & Dewey E. T. Epizootic fox encephalitis. I. General description. Am. J. Hyg. 12, 109–129 (1930).

[b3] Decaro N., Martella V. & Buonavoglia C. Canine adenoviruses and herpesvirus. Vet. Clin. North Am. Small Anim. Pract. 38, 799–814 (2008). 18501279 10.1016/j.cvsm.2008.02.006 PMC7114865

[b4] Walker D. . Infectious canine hepatitis in red foxes (*Vulpes vulpes*) in wildlife rescue centres in the United Kingdom. Vet. Rec. 178, 421 (2016). 27001767 10.1136/vr.103559

[b5] Cabasso V. J. Infectious canine hepatitis virus. Ann. N. Y. Acad. Sci. 101, 498–514 (1962). 14017542 10.1111/j.1749-6632.1962.tb18891.x

[b6] Gerhold R. W. . Infectious canine hepatitis in a gray fox (*Urocyon cinereoargenteus*). J. Wildl. Dis. 43, 734–736 (2007). 17984271 10.7589/0090-3558-43.4.734

[b7] Thompson H. . Infectious canine hepatitis in red foxes (*Vulpes vulpes*) in the United Kingdom. Vet. Rec. 166, 111–114 (2010). 20097890 10.1136/vr.b4763

[b8] Pursell A. R., Stuart B. P., Styer E. & Case J. L. Isolation of an adenovirus from black bear cubs. J. Wildl. Dis. 19, 269–271 (1983). 6315971 10.7589/0090-3558-19.3.269

[b9] Park N. Y., Lee M. C., Kurkure N. V. & Cho H. S. Canine adenovirus type 1 infection of a Eurasian river otter (*Lutra lutra*). Vet. Pathol. 44, 536–539 (2007). 17606519 10.1354/vp.44-4-536

[b10] Truyen U., Müller T., Heidrich R., Tackmann K. & Carmichael L. E. Survey on viral pathogens in wild red foxes (*Vulpes vulpes*) in Germany with emphasis on parvoviruses and analysis of a DNA sequence from a red fox parvovirus. Epidemiol. Infect. 121, 433–440 (1998). 9825797 10.1017/s0950268898001319 PMC2809543

[b11] Garcelon D. K., Wayne R. K. & Gonzales B. J. A serologic survey of the island fox (*Urocyon littoralis*) on the Channel Islands, California. J. Wildl. Dis. 28, 223–229 (1992). 1318424 10.7589/0090-3558-28.2.223

[b12] Stephenson R. O., Ritter D. G. & Nielsen C. A. Serologic survey for canine distemper and infectious canine hepatitis in wolves in Alaska. J. Wildl. Dis. 18, 419–424 (1982). 6296469 10.7589/0090-3558-18.4.419

[b13] Balboni A. . Molecular epidemiology of canine adenovirus type 1 and type 2 in free-ranging red foxes (*Vulpes vulpes*) in Italy. Vet. Microbiol. 162, 551–557 (2013). 23201241 10.1016/j.vetmic.2012.11.015

[b14] Webbon C. C., Baker P. J. & Harris S. Faecal density counts for monitoring changes in red fox numbers in rural Britain. J. Appl. Ecol. 41, 768–779 (2004).

[b15] Taylor C. S. . Increased prevalence and geographic spread of the cardiopulmonary nematode *Angiostrongylus vasorum* in fox populations in Great Britain. Parasitology 142, 1190–1195 (2015). 26027539 10.1017/S0031182015000463

[b16] Parry H. B. Viral hepatitis of dogs (Rubarth’s disease). I. Clinical and pathological observations on a spontaneous epidemic. Vet. Rec. 62, 559–565 (1950). 10.1136/vr.62.38.359 14782379

[b17] Meredith A. L., Cleaveland S. C., Brown J., Mahajan A. & Shaw D. J. Seroprevalence of *Encephalitozoon cuniculi* in wild rodents, foxes and domestic cats in three sites in the United Kingdom. Transbound. Emerg. Dis. 62, 148–156 (2015). 23607769 10.1111/tbed.12091

[b18] Zimmer I. A. . Report of *Trichinella spiralis* in a red fox (*Vulpes vulpes*) in Northern Ireland. Vet. Parasitol. 159, 300–303 (2009). 19070433 10.1016/j.vetpar.2008.10.066

[b19] Learmount J. . First report of *Trichinella pseudospiralis* in a red fox in mainland Britain, Vet. Parasitol. 208, 259–262 (2015). 25659659 10.1016/j.vetpar.2015.01.011

[b20] Baker L. A., Jensen H. E. & Witter R. E. Canine infectious hepatitis – fox encephalitis. J. Am. Vet. Med. Assoc. 124, 214–216 (1954).

[b21] Morrison M. D., Onions D. E. & Nicolson L. Complete DNA sequence of canine adenovirus type 1. J. Gen. Virol. 78, 873–878 (1997). 9129661 10.1099/0022-1317-78-4-873

[b22] Crowther J. R. The ELISA Guidebook (Humana Press, 2000).

[b23] Robin X. . pROC: an open-source package for R and S+ to analyze and compare ROC curves. BMC Bioinform. 12, 77 (2011). 10.1186/1471-2105-12-77 PMC3068975 21414208

[b24] Core Team R.. R: The R Project for Statistical Computing. (Date of access: 01/06/16) Available at: https://www.r-project.org (2016).

[b25] Metz C. E. Basic principles of ROC analysis. Semin. Nucl. Med. 8, 283–298 (1978). 112681 10.1016/s0001-2998(78)80014-2

[b26] Lopéz-Ratón M., Rodríguez-Álvarez M. X., Cadarso-Suárez C. & Gude-Sampedro F. An R package for selecting optimal cutpoints in diagnostic tests. J. Stat. Softw. 61, 1–36 (2014).

[b27] Loeffen W. . Development of a virus neutralisation test to detect antibodies against Schamallenberg virus and serological results in suspect and infected herds. Acta Vet. Scand. 54, 44 (2012). 22871162 10.1186/1751-0147-54-44 PMC3503834

[b28] Harrell F. E., Lee K. L. & Mark D. B. Multivariable prognostic models: issues in developing models, evaluating assumptions and adequacy, and measuring and reducing errors. Stat. Med. 15, 361–387 (1996). 8668867 10.1002/(SICI)1097-0258(19960229)15:4<361::AID-SIM168>3.0.CO;2-4

[b29] Hosmer D. W. & Lemeshow S. Assessing the fit of the model. In Applied Logistic Regression 2^nd^ edn (eds Hosmer, & Lemeshow, ) Ch. 5, 143–202 (John Wiley & Sons, 2000).

[b30] Isaaks E. H. & Srivastava R. M. Applied Geostatistics (eds Isaaks E. H. & Srivastava R. M.) (Oxford University Press, 1989).

[b31] Wickham H. ggplot2: Elegant graphics for data analysis (Springer-Verlag, 2009).

[b32] Bivand R. . rdgal: bindings for the geospatial data abstraction library. R package version 1.1-10. (Date of access: 02/06/16) Available at: https://cran.r-project.org/package=rgdal (2016).

[b33] Global Administrative Areas: GADM Database of Global Administrative Areas. (2015) (Date of access: 23/02/16) Available at: www.gadm.org.

[b34] Bivand R. . maptools: tools for reading and handling spatial objects. R package version 0.8-39. (2016) (Date of access: 02/06/16) Available at: https://cran.r-project.org/package=maptools.

[b35] Hijmans R. J. . raster: geographic data analysis and modelling. R package version 2.5-2. (2015) (Date of access: 02/06/16) Available at: https://cran.r-project.org/package=raster.

[b36] Mazerolle M. J. AICcmodavg: model selection and multimodel inference based on (Q)AIC(c). R package version 2.0-4. (2016) (Date of access: 02/06/16) Available at: https://cran.r-project.org/package=AICcmodavg.

[b37] Dardis C. LogisticDx: diagnostic tests for models with a binomial response. R package version 0.2. (2015) (Date of access: 02/06/16) Available at: https://cran.r-project.org/package=LogisticDx.

[b38] Ribeiro P. J. Jr. & Diggle P. J. geoR: a package for geostatistical analysis. R News. 1, 14–18 (2001).

[b39] Böhm M. . Serum antibody titres to canine parvovirus, adenovirus and distemper virus in dogs in the UK which had not been vaccinated for at least three years. Vet. Rec. 154, 457–463 (2004). 15119729 10.1136/vr.154.15.457

[b40] Baker P. J., Robertson C. P. J., Funk S. M. & Harris S. Potential fitness benefits of group living in the red fox, Vulpes vulpes. Anim. Behav. 56, 1411–1424 (1998). 9933538 10.1006/anbe.1998.0950

[b41] Åkerstedt J. . Serosurvey for canine distemper virus, canine adenovirus, *Leptospira interrogans*, and *Toxoplasma gondii* in free-ranging canids in Scandinavia. J. Wildl. Dis. 46, 474–480 (2010). 20688639 10.7589/0090-3558-46.2.474

[b42] Almberg E. S., Mech L. D., Smith D. W., Sheldon J. W. & Crabtree R. L. A serological survey of infectious disease in Yellowstone National Park’s canid community. PLoS One 4, e7042 (2009). 19756151 10.1371/journal.pone.0007042 PMC2738425

[b43] Fox J. P., Hall C. E. & Cooney M. K. The Seattle Virus Watch VII: observations of adenovirus infections. Am. J. Epidemiol. 105, 362–386 (1977). 192073 10.1093/oxfordjournals.aje.a112394

[b44] Roy S. . Isolation and characterization of adenoviruses persistently shed from the gastrointestinal tract of non-human primates. PLoS Pathogens 5, e1000503 (2009). 19578438 10.1371/journal.ppat.1000503 PMC2698151

[b45] Rowe W. P., Huebner R. J., Gilmore L. K., Parrott R. H. & Ward T. G. Isolation of a cytopathogenic agent from human adenoids undergoing spontaneous degeneration in tissue culture. Proc. Soc. Exp. Biol. Med. 84, 570–573 (1953). 13134217 10.3181/00379727-84-20714

[b46] Garnett C. T., Erdman D., Xu W. & Gooding L. R. Prevalence and quantitation of species C adenovirus DNA in human mucosal lymphocytes. J. Virol. 76, 10608–10616 (2002). 12368303 10.1128/JVI.76.21.10608-10616.2002 PMC136639

[b47] Kojaoghlanian T., Flomenberg P. & Horwitz M. S. The impact of adenovirus infection on the immunocompromised host. Rev. Med. Virol. 13, 155–171 (2003). 12740831 10.1002/rmv.386

[b48] Walls T., Shankar A. G. & Shingadia D. Adenovirus: an increasingly important pathogen in paediatric bone marrow transplant patients. Lancet Infect. Dis. 3, 79–86 (2003). 12560192 10.1016/s1473-3099(03)00515-2

[b49] Balboni A. . Investigation of the presence of canine adenovirus (CAdV) in owned dogs in Northern Italy. Res. Vet. Sci. 97, 631–636 (2014). 25468801 10.1016/j.rvsc.2014.10.010

[b50] Nilsen E. B. . Wolf reintroduction to Scotland: public attitudes and consequences for red deer management. Proc. Biol. Sci. 274, 995–1002 (2007). 17264063 10.1098/rspb.2006.0369 PMC2141678

[b51] Wilson C. J. Could we live with reintroduced large carnivores in the UK? Mammal Rev. 34, 211–232 (2004).

[b52] Reis S. . UK gridded population based on Census 2011 and Land Cover Map 2007. NERC Environmental Information Data Centre. (2016) (Date of access: 21/05/16) Available at: http://doi.org/10.5285/61f10c74-8c2c-4637-a274-5fa9b2e5ce44.

[b53] Perry M. & Hollis D. The generation of monthly gridded datasets for a range of climatic variables over the UK. Int. J. Climatol. 25, 1041–1054 (2005).

[b54] Jarvis A., Reuter H. I., Nelson A. & Guevara E. Hole-filled seamless data V4, International Centre for Tropical Agriculture (CIAT) (2008) (Date of access: 21/05/16) Available at: http://srtm.csi.cgiar.org.

[b55] European Environment Agency. Corine land cover (CLC) 2012 Version 18.5. (2012) (Date of access: 21/05/16) Available at: http://land.copernicus.eu.

