# Supplementary material for: Serological and molecular epidemiology of canine adenovirus type 1 in red foxes (Vulpes vulpes) in the United Kingdom
Source: Sci Rep. 2016 Oct 31;6:36051. doi: 10.1038/srep36051 (PMC5086850; doi:10.1038/srep36051)
Supplement: Supplementary Information [file srep36051-s1.pdf]

Supplementary Information

Serological and molecular epidemiology of canine adenovirus type 1 in red foxes (*Vulpes vulpes*) in the United Kingdom

David Walker [a], Seán A. Fee [b], Gill Hartley [c], Jane Learmount [d], Maria J.H. O'Hagan [e], Anna L. Meredith [a], Barend M. de C. Bronsvoort [a], Thibaud Porphyre [a], Colin P. Sharp [a], Adrian W. Philbey [a]

[a] *Royal (Dick) School of Veterinary Studies and The Roslin Institute, University of Edinburgh, Easter Bush, Edinburgh EH25 9RG, UK*
[b] *Veterinary Sciences Division, Agri-Food and Biosciences Institute, Beltany Road, Coneywarren, Omagh BT78 5NF, UK*
[c] *Science and Advice for Scottish Agriculture, Roddinglaw Road, Edinburgh EH12 9FJ, UK*
[d] *National Wildlife Management Centre, Animal and Plant Health Agency, Sand Hutton, York YO41 1LZ, UK*
[e] *Veterinary Epidemiology Unit, Department of Agriculture, Environment and Rural Affairs, Upper Newtownards Road, Belfast BT4 3SB, UK*

Availability of materials and data

In Supplementary Dataset S1, the location data for red foxes are anonymised to UK 'vice county' level for data protection reasons. Identifiable clinical data from the dogs and red foxes from veterinary hospitals (i.e. case numbers, owner name) have also been anonymised or removed from both Supplementary Dataset S1 and Supplementary Dataset S2.

Supplementary Table 1. Summary of canine adenovirus type 1 (CAV-1) PCR screening results of samples from red foxes determined to be positive for CAV-1 in liver and/or kidney. All other foxes subjected to molecular testing were negative for CAV-1 in liver and kidney by PCR.

Fox ID	Liver	Kidney	Blood	Spleen	Brain	Lung	GIT	Urine	Faeces
061014/2	1	0	0	0	0	-	-	0	-
111114/1	1	0	1	-	1	0	-	0	-
201114/1	1	1	1	-	1	-	-	-	0
201114/2	1	1	1	1	1	1	-	-	0
120115/1	1	0	-	-	-	-	-	-	-
120115/3	1	0	-	-	-	-	-	-	-
300115/1	1	0	0	-	-	0	-	-	-
300115/2	1	1	0	-	-	1	-	1	-
300115/3	0	1	-	-	-	-	-	-	-
020215/1	0	0	0	1	1	0	0	-	0
090315/1	1	1	0	1	0	0	0	1	0
090315/2	1	1	1	-	-	0	-	1	-
030415/1	1	0	0	1	1	0	0	0	0
010515/5	0	1	0	-	-	-	-	-	-
220515/1	1	0	1	1	0	1	0	-	0
15195	1	0	-	-	-	-	-	-	-
15346	0	1	0	-	-	-	-	-	-
15620	1	1	0	-	-	-	-	-	-
15622	1	0	0	-	-	-	-	-	-
15703	0	1	0	-	-	-	-	-	-
15705	0	1	0	-	-	-	-	-	-
16036	1	0	-	-	-	-	-	-	-
16137	0	1	-	-	-	-	-	-	-
16185	1	0	-	-	-	-	-	-	-
16432	0	1	0	-	-	-	-	-	-
16606	1	0	0	-	-	-	-	-	-
17066	1	1	0	-	-	-	-	-	-
17154	1	1	1	-	-	-	-	-	-
17157	1	0	0	-	-	-	-	-	-

1, positive for CAV-1; 0, negative for CAV-1; -, sample not available; GIT, gastrointestinal tract
